# To the Editors of the Medical and Physical Journal

**Published:** 1805-04-01

**Authors:** Edward Luscombe

**Affiliations:** King's Barracks, Canterbury


### *To the Editors of the Medical and Physical Journal.*

GENTLEMEN,

As you have frequently expressed a wish, that in the event of meeting with any epidemic disease, I would make known to you the circumstances attending it; I lose no time in acquainting you, that (in the absence of the surgeons of the corps) the Deputy Inspector of Hospitals consigned to my care on the 11th of January, the hospital of the three troops of the Waggon Train stationed at Canterbury; which at this time contained such cases only as are always to be met with in military hospitals. But on the 4th of February, a man was admitted with fever; on the 9th three men with fever were admitted; on the 12th, two; 13th, one; 21st, three; 22d, one; exclusive of three non-commissioned officers attacked between the 15th and 21st, being nearly one-tenth part of the men present.

Those men were attacked in a manner nearly similar; the disease was ushered in with severe rigors, loss of appetite, disturbed sleep, and nausea. They complained principally of pain in the head, loins, and extremities; thirst; and weakness. On their admission the pulse was generally quick but weak; the tongue white or furred; the heat of the body was frequently not increased, although in most cases it *soon* became much greater than in health. Derangement of the biliary system was very striking; and in several cases very large quantities of bile were evacuated.

On first seeing the patient I invariably directed that an emetic should be immediately given, and it was most commonly followed by six grains of calomel; if the heat of the body became greater than in health and permanent, cold affusion was had recourse to; if much fever remained after this, with increased action of the heart and arteries with augmented power, antimonial powder (together with calomel till the primæ viæ were cleared of bile) was prescribed; in other cases an eighth of a grain of tartarized antimony, eight drops of tincture of opium with half an ounce of aq. ammon. acetat. and the same quantity of water, were given every four hours. By these means I was enabled in most cases to cut short the disease; but in two or three instances it went on to the second stage. In two of those cases (one of which was accompanied with almost incessant diarrhœa) calomel appeared to produce the most beneficial effects; wine and brandy were given *freely*, (sometimes

(sometimes every hour) when required by the state of the pulse, &c. On the decline of the disease decoction of bark acidulated with sulphuric acid or infusion of quassia was given; and the men were taken out in a spring waggon for three or four hours in every day. *All my patients* are now convalescents, or have returned to their duty; except one man in whom the fever has become intermittent, as happened in the case of one of the non-commissioned officers.

No fresh cases have occurred since the 22d of February, and I trust the progress of the disease has been arrested. The hospital was fumigated with the mineral acid gases several times in a day; the wards in which there were men at the time, with muriatic acid gas; and the rooms which were at the moment empty, with oxygenated muriatic acid gas.

Cleanliness and the free admission of fresh air were insisted on.

The crowded barrack-rooms I had ventilated, so as to allow the impure air to escape without difficulty; and to secure a supply of air more fit for the purposes of respiration. The whole of the bedding was at my request exposed to the open air for several hours in the day.

It may be right to remark, that for some days after the last case of fever was admitted, several men were daily attacked with ophthalmia, which appeared to have been epidemic in the 18th and 53d regiments, which were a short time since stationed at Canterbury.

I am, &c.

EDWARD LUSCOMBE.

*King's Barracks, Canterbury, March 2,* 1805.

---

